# Exploring Political Views on Synthetic Biology in the Netherlands

**DOI:** 10.1007/s11569-016-0257-2

**Published:** 2016-04-22

**Authors:** Virgil Rerimassie

**Affiliations:** Rathenau Instituut, PO Box 95366, 2509 CJ The Hague, The Netherlands

**Keywords:** Synthetic biology governance, Deliberative democracy, Parliamentary technology assessment, Public engagement, Public dialogue

## Abstract

Synthetic biology may be an important source of progress as well as societal and political conflict. Against this backdrop, several technology assessment organizations have been seeking to contribute to timely societal and political opinion-making on synthetic biology. The Rathenau Instituut, based in the Netherlands, is one of these organizations. In 2011, the institute organized a ‘Meeting of Young Minds’: a young people’s debate between ‘future synthetic biologists’ and ‘future politicians’. The former were represented by participants in the international Genetically Engineered Machines competition (iGEM), the latter by political youth organizations (PYOs) linked to Dutch political parties. The Rathenau Instituut found seven PYOs—including right wing, left wing, Green and Christian groups—willing to commit to an intensive process aimed at formulating a tentative partisan view on synthetic biology and discussing it with fellow PYOs and iGEM participants. Given the minimal amount of available data on how political parties understand synthetic biology, mapping the debate may provide valuable insights. In this article, I aim to provide such a mapping exercise and also to reflect on how and why the Rathenau Instituut organized the event.

## Introduction

*The commercial use of synthetic biology poses significant threats to the earth*’*s biodiversity*, *could speed rainforest destruction by increasing demand for sugar*, *and harm sustainable farmers and poor communities across the world whose cultures and income depend on farming truly natural commodities such as coconut oil* [1].

This quote is part of a petition called ‘Synthetic biology is not natural. Keep extreme genetic engineering out of ‘natural’ products’, launched by the ETC Group, Friends of the Earth and a number of other NGOs in the summer of 2014. These NGOs have been voicing their critique on synthetic biology—the latest phase in the development of biotechnology—for a couple of years now [2]. Nevertheless, this particular petition is symbolic of the current state of the art in the development of synthetic biology. It was a response to the news that the Belgian company Ecover—dedicated to the development of natural cleaning products—intended to develop a soap containing oil produced from algae whose genetic code had been altered using synthetic biology, to make it suitable for use in closed fermentation facilities, for example. According to Ecover, the oil is a sustainable and natural alternative to palm kernel oil, which is an important cause of deforestation of tropical rain forests [3]. Not long after Ecover’s announcement, the multinational Unilever announced the development of a similar product [4]. Synthetic biology is thus slowly leaving the laboratory phase, and products made by means of synthetic biology are gradually entering the consumer market.

In spite of the important benefits synthetic biology may offer, such as in the fields of medicine and sustainability, its development is certainly not welcomed by everyone, as the petition illustrates. Synthetic biology also gives rise to concerns about potential environmental and health risks [5, 6]. At the same time, the field raises difficult moral questions, since it allows scientists to consider living organisms in an unprecedented manner [7, 8]. Indeed, similar to the debates triggered by Dolly the cloned sheep and the—still unsettled—controversies regarding genetically modified foods [9, 10], synthetic biology may be a source of tension and conflict.

In order to understand the potential issues raised by synthetic biology in a timely manner and contribute to shaping the field, many organizations and academic groups involved in technology assessment (TA) and the examination of the ethical, legal and societal implications of emerging technologies (ELSI) engaged with the field early on in its development. This early activity corresponds with the shift in the focus of TA towards more ‘early engagement’ in the last 10 years [e.g. 11, 12] and the move ‘upstream’ in the innovation process, that is, from the final products to the sources of innovation in research and development processes [13]. These efforts have culminated in comprehensive analyses of the ethical, legal and societal implications and questions synthetic biology may give rise to [e.g. 6, 14, 15]. For instance, are the potential risks outweighed by the potential benefits? Who profits from these developments and who carries the burden? Are synthetic biologists overstepping moral boundaries that should not be overstepped? In the end, these are *political* and *societal* questions and therefore eventually need to be answered by politics and society [16]. As the aforementioned petition indicates, societal debates on synthetic biology are slowly taking form. Yet, up to now, these remain rather modest, which is not surprising since awareness of synthetic biology is still rather low [17]. How the questions raised by synthetic biology will be answered by politics and society therefore largely remains to be seen.

This image resonates well with the situation in the Netherlands. The Rathenau Instituut—the Dutch office for technology assessment and science system assessment—engaged with synthetic biology early on and undertook various activities to bring synthetic biology into societal and political debate [for an overview, see 16]. In this article, I aim to explore political views on synthetic biology in the Netherlands. In order to do so, I discuss the results of one of these activities: the so-called Meeting of Young Minds (MOYM). This event encompassed a young people’s debate on synthetic biology between ‘future politicians’, represented by members of Dutch political youth organizations (PYOs) and ‘future synthetic biologists’, represented by participants in the international Genetically Engineered Machines competition, better known under its acronym ‘iGEM’. In the absence of a political discussion to date of the writing of this article, the intention is to provide empirical data on the possible viewpoints of Dutch political parties on synthetic biology.

The article is structured as follows: First, I will provide some background information and explain the context in order to point out *why* and *how* the Rathenau Instituut organized the Meeting of Young Minds debate. Accordingly, I will briefly discuss the role and position of the institute and describe some of its efforts to bring synthetic biology into political and societal debate. Second, I will discuss the rationale of reaching out to PYOs to facilitate political engagement with synthetic biology and briefly describe the participating PYOs. Next, I will elaborate on the preparation for (including how the Rathenau Instituut supported the political opinion-making process of the PYOs) and the organization and execution of the debate. The remainder of the article is dedicated to mapping viewpoints expressed during the Meeting of Young Minds debate. To this end, a framework developed in the project Global Ethics in Science and Technology (GEST) will be applied to structure the results [18]. Following the application of this framework, discourses on *innovation*, *risk*, *broader ethical issues* and *power and control* will be used as lenses through which the debate can be examined and the issues that are raised can be highlighted.

## Efforts of the Rathenau Instituut to Foster Political Opinion-Making on Synthetic Biology

In order to better understand the MOYM debate, I will first discuss how the event came to be held. Accordingly, I will briefly discuss the formal role of the Rathenau Instituut and place the event in the context of the institute’s earlier activities to bring synthetic biology into societal and political debate.

### Role and Position of the Rathenau Instituut

The Rathenau Instituut functions as the Dutch office for technology assessment and science system assessment. The formal description of the technology assessment task of the institute reads as follows:

[t]he role of the institute is to contribute to societal debate and the formation of political opinion on issues that relate to or are the consequence of scientific and technological developments. This specifically includes the ethical, social, cultural and legal aspects of such developments. In particular, the institute facilitates the formation of political opinion in both chambers of the Parliament of the Netherlands and in the European Parliament. [19, derived from 20]

The institute’s broad technology assessment task is to stimulate societal and political debate on (emerging) science and technology. This includes—but certainly is not limited to—TA specifically aimed at stimulating parliamentary debate on the role of science and technology in society. In this regard, the position of the institute should be taken into account. As Ganzevles et al. [21] point out, the position of the institute significantly influences how a given TA organization operates towards the spheres of parliament, government, science and technology, and society. For instance, the Parliamentary Office of Science and Technology (POST) in the UK is situated inside parliament and works in close contact with Members of Parliament. In contrast, while parliament and the government are its main clients, the Rathenau Instituut is more distant from the political process and has an autonomous position. This independent position vis-à-vis the government and parliament allows the institute the freedom to determine its strategies and working plan but does not guarantee an audience [16, 20]. This has clearly influenced the institute’s activities with regard to synthetic biology.

### Engaging with Synthetic Biology

The Rathenau Instituut engaged with synthetic biology quite early on in its development. The institute had a longstanding interest in the development of biotechnology, but synthetic biology was a particular focus for the institute due to its interest in NBIC convergence, the synergetic convergence of nanotechnology, biotechnology, ICT and cognitive sciences [22]. Due to the increasing number of people attracted to this field and the growing number of publications and review articles in scientific journals with regard to synthetic biology, the Rathenau Instituut assessed that there ‘was something happening’ [23]. In 2006, a researcher from the Rathenau Instituut attended the Synthetic Biology 2.0 conference in Berkeley, CA. The experience served as one of the major sources of inspiration for the report *Constructing Life* [23], which was one of the first reports concerned with the potential societal impact of synthetic biology. In 2007, the institute published a Dutch version of the report [24] and a *Message to the Parliament* based thereon [25], a brief summary of the study and recommendations. As a result of these efforts, members of the Dutch Labour Party (Partij voor de Arbeid) raised questions in the Dutch parliament to draw the attention of the cabinet to synthetic biology [26]. In its response, the cabinet underscored the importance of monitoring the developments in the field and, for instance, requested the Commission on Genetic Modification to do so [27]. Yet, during the next 5 years, synthetic biology did not become a topic of debate in parliament. This is perhaps not surprising, since—in spite of important scientific breakthroughs in the field—synthetic biology is still largely confined to the laboratory and concrete applications largely remain absent. During this period, the Rathenau Instituut closely monitored the developments in the field and participated in international projects dedicated to analysing the potential impact of synthetic biology, such as *Synthetic Biology for Health*, *Ethical and Legal Issues* (SYBHEL 2009–2012) [16]. In addition, synthetic biology played an important part in activities dedicated to NBIC convergence. In this context, the institute published the book *Life as a Construction Kit* [28], launched during the Dutch Societal Dialogue on Nanotechnology (Maatschappelijke Dialoog over Nanotechnologie), which was organized by an independent committee by order of the Dutch government.^1^

## Looking for Novel Approaches to Facilitate Political Engagement

From 2006, when *Constructing Life* was published, synthetic biology continued to develop. The most famous scientific breakthrough is perhaps the creation of a bacterium with a fully synthetic genome by the group led by Craig Venter [30]. In addition, more groups became active in the field and more investments were made [31]. During this period, many scholars, advisory committees and (TA) organizations also explored and deepened the various ethical, legal, societal and risk-related questions that synthetic biology can raise. Zhang, Marris and Rose [32] identified no less than 39 different reports written in English from 2004 to 2011. According to Calvert and Frow [33], these reports raise a fairly consistent set of issues related to synthetic biology: How do we deal with biosafety and biosecurity risks? How do we organize intellectual property? Are there any (moral) limits to ‘creating life’ that should not be overstepped? And how do we involve the public in the development of the field? Although these tough questions are posed by the academia and advisory committees, they are *societal and political questions* and thus are in need of *societal and political answers*. Moreover, the sort of questions raised by synthetic biology cannot always self-evidently be answered with reference to established political ideologies. As the case of Ecover illustrates, synthetic biology may contribute to sustainability, but (for some) this is at the expense of ‘naturalness’. This tension is particularly problematic for Green-oriented parties that value both naturalness and sustainability and are used to them going hand in hand rather than having to choose one at the expense of the other. It is certainly true that the questions raised by synthetic biology may cause political and societal tensions.

Given the pace of the development of the field and its potential for controversy, the Rathenau Instituut sought to further politicize and democratize [34] synthetic biology. From 2011, almost 5 years after the publication of *Constructing Life*, the Rathenau Instituut intended to broaden the debate on synthetic biology again. In the succeeding years, the institute undertook various engagement activities in different spheres of the science and technology governance landscape [16]. In the societal sphere (civil society and the general public), for instance, the institute co-organized a workshop on how synthetic biology challenges ‘symbolic order’ [cf. 28, 29] in collaboration with the Dutch Foundation for Christian Philosophy [35]. In addition, it contributed to several initiatives aimed at informing the general public, such as a quarterly educational magazine dedicated to the life sciences [36] and the popular Dutch science communication website Kennislink [37]. Furthermore, the institute undertook several activities in the science and technology sphere (university or industry researchers and technology developers), such as presenting at several national and international conferences on synthetic biology [16]. Lastly, the political sphere (not only primarily parliament but also ministries and other government agencies) is of particular importance for the institute given its formal task. However, because the field is still in the experimental stage of development and because of the lessons learned from recent experiences of the political debate on nanotechnology [38], the institute expected that Members of Parliament would be unlikely to prioritize synthetic biology over other more urgent issues. Therefore, the institute did not consider the time right to encourage a parliamentary debate on synthetic biology and started to look for novel approaches to facilitate political awareness and discussion on synthetic biology.

### The Role of iGEM

One clear sign of the growth of the field of synthetic biology is the immense popularity of iGEM, the international Genetically Engineered Machines competition. In this competition, students use standardized and interchangeable genetic building blocks (BioBricks™) to design microorganisms with novel and useful properties [39]. iGEM began in 2003 as a summer course for students at the Massachusetts Institute of Technology. In 2004, the course was transformed into a competition in which five different teams participated. In 2011, the competition had grown into a full-blown international competition, in which no less than 160 teams participated from 30 countries [39]. In spite of having limited means and a short timeframe, the projects are often impressive. Due to the popularity of the competition, the iGEM Foundation decided in 2011 to organize three regional preliminaries (or ‘jamborees’ in iGEM jargon). The European–African Jamboree was to be held in Amsterdam, which provided the Rathenau Instituut with a good opportunity to broaden the so far modest political discussion on synthetic biology.

### Reaching Out to Political Youth Organizations

This large gathering of young synthetic biologists sparked the idea of organizing a young people’s debate on synthetic biology: a Meeting of Young Minds involving a debate between future synthetic biologists and future politicians. The iGEM teams were addressed as future synthetic biologists. In this regard, the so-called policy and practices (previously called human practices) element has been of great importance. This implies that the iGEM participants do not only work on their project in the laboratory but also need to pay close attention to the societal aspects of their research and to reach out to society. The idea of a MOYM therefore resonated well with the culture of iGEM, and the organization was very willing to cooperate [16, 39].

The future politicians were sought in the circles of Dutch political youth organizations (PYOs). PYOs are organizations tied to a specific political party that are open to membership for young people between approximately 14 and 27 years old. PYOs aim to promote and maintain the causes of their political party by a variety of means, such as participating in debates, initiating petitions or organizing publicity stunts [40].^2^ Although they are affiliated with a specific political party, most PYOs are independent and are therefore allowed to form a dissenting opinion. In this sense, they also act as an important internal checks-and-balances instrument for political parties. PYOs often have impressive membership numbers and are seen as an important breeding ground for future politicians. In fact, many prominent Dutch politicians were active in a PYO, such as Mark Rutte, the current prime minister [41]. In 2011, ten political parties were represented in the Dutch parliament. All of them, with the exception of the Partij voor de Vrijheid (whose leader is Geert Wilders), were affiliated with a PYO. The Rathenau Instituut found that seven of these PYOs were willing to formulate a tentative political view on synthetic biology and enter into a debate with each other and representatives from iGEM. The institute did not succeed in mobilizing ROOD, a socialist PYO that is connected to the SP, the Dutch Socialist Party and the JOVD, which is linked to the (moderate right wing) VVD, the People’s Party for Freedom and Democracy. A brief characterization of the seven PYOs that did participate is found in Table 1. The characterization does not do justice to the richness and complexity of the ideologies of the PYOs and their ‘mother parties’ but nevertheless provides readers who are unfamiliar with the (diverse) Dutch political landscape with a useful overview.

**Table 1:** Characterization of PYOs

PYO	Affiliation	Ideology
Christen Democratisch Jongeren Appèl (CDJA)	Christen Democratisch Appèl (CDA)	Christian democratic, conservative, centre-right wing [42]
DWARS	GroenLinks	Green, left wing [43]
Jonge Democraten	Democraten 66 (D66)	Social liberalistic [44]
Jonge Socialisten	Partij van de Arbeid	Labour, social democratic [45]
PerspectieF	ChristenUnie	Social Christian [46]
PINK!	Partij voor de Dieren	Animal-welfare driven, Green [47]
SGP-jongeren (SGPJ)	Staatkundig Gereformeerde Partij (SGP)	Radical Christian (Protestant), conservative [48]

As the table demonstrates, a total of seven participating PYOs demonstrate a high degree of pluralism, varying from right wing to left wing and from animal-welfare inspired to Christian faith-based.

## Organizing the Meeting of Young Minds

In this section, I will discuss the steps the Rathenau Instituut took to prepare the MOYM debate, such as the different capacity-building actions. I will also provide further details about how the institute organized the event.

### Preparation and Capacity-Building

As already mentioned, seven PYOs were willing to participate. The institute contacted the PYOs via their board secretaries, who forwarded the request to participate in the upcoming activities to active members of the PYO who seemed fit to undertake this task (and could decide on participation). Eventually, small ad hoc working groups were formed consisting mostly of board members, members of pre-existing working groups (e.g. on sustainability and health) or a combination of both. In all cases, the representatives were mandated to act on behalf of their PYO.

The first step the institute took after getting in touch with the PYO representatives was organizing a kick-off event. During this event, researchers from the institute and former iGEM participants provided a general introduction about synthetic biology and the academic discussion so far. Furthermore, the institute presented its ideas on the event and supporting actions, and luckily these received a positive response.

It is important to note that the participating PYOs barely had any knowledge about synthetic biology. Therefore, the institute undertook several actions to support the PYOs in their opinion-making process on synthetic biology. There is a clear risk of framing synthetic biology in a particular manner, and thus the Rathenau Instituut needed to ensure that it provided a balanced view of the developments. In order to do so, first of all, a selection of various sorts of reports dedicated to synthetic biology was made, such as not only those from the European Group on Ethics [6], the (UK) Royal Academy of Engineering [49], the Biotechnology and Biological Sciences Research Council [50] and the (US) Presidential Commission for the Study of Bioethical Issues [51] but also the concerned ETC Group [52]. Next, an expert meeting was organized with the iGEM team of the Technical University of Delft [53, 54]. At this meeting, researchers from the institute also aimed to provide multiple views on synthetic biology. Some of the experts stemmed from the field of synthetic biology itself (such as the Dutch iGEM teams, which presented their projects), but others had risk assessment, intellectual property or philosophy perspectives. Lastly, in collaboration with scholars Tsjalling Swierstra and Marianne Boenink, techno-moral vignettes on synthetic biology were developed.^3^ These are brief ‘snapshots’ of a future situation in which synthetic biology is applied but at the same time raises moral questions. Rather than being predictions that close the debate, they are designed to be invitations to come up with imaginations of how science and technology could improve our lives [55, 56].

### Organization of the Event

The institute thus provided several supporting actions to aid the PYOs in their opinion-making on synthetic biology. How the (tentative) views on synthetic biology were formed was left to the PYOs themselves, according to their own internal procedures. In retrospect, this took place within the ad hoc working groups (consisting of about five PYO representatives).

In terms of the event itself, the institute chose a debate format, rather than, for instance, an exercise aimed at letting participants work towards a common vision of a technological future in which synthetic biology plays an important role. Given the absence of a mature political discussion in the Netherlands, the institute was primarily motivated to broaden the debate on synthetic biology with reference to (non-neutral) partisan perspectives. It assumed that a debate format would serve this goal best, not least because an exchange of arguments would lead to a deeper understanding of the different perspectives on synthetic biology. At the same time, the institute hoped that the event would also promote mutual learning and understanding among the PYOs and iGEM participants.

In order to prepare for the debate, the institute asked the PYOs to draft a ‘political pamphlet’, a two-page document in which they outlined their general views on synthetic biology. In addition, they were asked to supplement the document with position statements in order to specify their views and provide input on how to organize the debate. Other than that, the institute had no specific demands or questions. In most cases, these pamphlets were the results of deliberation within the ad hoc working groups. Two PYOs, however, went further in this regard. The Christian Democratic CDJA, for instance, adopted an official resolution on synthetic biology following its internal procedures [57]. Similarly, the Green PYO DWARS adopted a so-called vision statement after consulting members during an internal discussion evening [58]. For the debate itself, the institute asked the PYOs to appoint one spokesperson who would represent them.

Due to time constraints, researchers from the institute could not engage with the iGEM teams as intensively as they had with the PYOs (when the trajectory with the PYOs started, the iGEM competition activities had already been going on for a while). Instead, together with the European iGEM committee, the institute made contact with iGEM teams that seemed to be excelling in their human practices and outreach activities, namely the teams from Imperial College London, University College London, the University of Potsdam, Paris Descartes University, the University of Freiburg and the University of Leuven. Since the institute contacted these teams late in the process, they were merely asked to appoint a spokesperson who would represent them during the debate. In spite of their representatives’ prior involvement in the process, the institute assumed that they could play an important role during the debate, given their (relative) expertise in synthetic biology and remarkable human practices activities.

The valuable information in the political pamphlets would be used to initially structure the debate. Three core themes on which the PYOs seemed to disagree were identified: promises, regulation and ownership. The plan was for each of the themes to be discussed in three rounds of 30 min. A researcher from the Rathenau Instituut with experience in moderating discussions was to lead the debate. In terms of the format of the debate (after a brief introduction of the theme), each round would start off by asking two opposing PYOs to move to the centre of the stage, defend a position statement and react to each other’s arguments.^4^ Next, the other PYOs (which would be located on the right-hand side of the stage) could join in the discussion. In order to do so, they would position themselves behind an interruption microphone, similar to those used in Dutch parliamentary debates. Lastly, the representatives from iGEM (located on the left-hand side of the stage) would be able to join in the discussion, also by means of an interruption microphone. The researchers from the institute planned to allow a bit more time for the PYOs than the iGEM teams because the iGEM teams had far less preparation time. Also, the institute wanted to increase the exposure of the—so far fairly unknown—political (partisan) views on synthetic biology. Nevertheless (also due to the moderator), the iGEM teams were certainly able to make a significant contribution to the discussion, as can be seen in the next section.

The MOYM debate took place in the grand auditorium of the VU University Amsterdam, on the night preceding the 2011 European–African iGEM Jamboree. It was open to the public and attended by about 350–400 visitors. The majority of the attendees consisted of iGEM participants.

## Mapping the Meeting of Young Minds

In the previous section, I outlined why and how the Rathenau Instituut engaged with Dutch PYOs and the iGEM community to broaden the modest political debate on synthetic biology in the Netherlands. In this section, I will map the results of the MOYM debate.^5^ The mapping exercise will be primarily based on excerpts from the MOYM debate. The political pamphlets provide valuable additional material, especially because it is unlikely that the debate allowed enough time for all of the viewpoints to be discussed. Unless indicated otherwise, the quotations used later in this article stem from the MOYM debate.

In order to structure the results of the debate and the content of the political pamphlets, I will draw from a (slightly altered) framework developed in the EU project Global Ethics on Science and Technology (GEST) [18]. The aim of this project was to better understand the ways in which expectations, tensions and conflicts surrounding science and technology relate to the specifics of different fields and to the broader societal contexts. In the framework developed to this end, the emphasis is on societal discourses as central storylines in discussions on science and technology [62]. The framework thus allows a systematic mapping of expectations, tensions and conflicts arising, or potentially arising, from developments in science and technology. It has been proven to be useful to analyse and compare discourses on emerging technologies—such as synthetic biology and nanotechnology—in Europe, China and India [63, 64]. Drawing from this framework, four different discourses will be used as lenses through which the issues relating to *innovation*, *risk*, (*broader*) *ethical issues* and *power and control* can be highlighted.^6^

Lastly, I will briefly and cautiously reflect on how the positions of the PYOs relate to the position of their mother parties towards (earlier) biotechnologies. However, it should be noted that comparison is not straightforward, because for the last decade, the Dutch political debate on biotechnology has been heavily focused on GMOs in agriculture and the specific dynamics of these applications [cf. 65].

### Innovation Discourse

Why is synthetic biology important? What kind of potential benefits does the field offer and what is needed to realize those benefits? These are the kind of questions that play a role in an innovation discourse. Such questions certainly played a role during the MOYM debate.

Among all of the participating PYOs—also among those that turned out to be critical—the institute saw an acknowledgement of the potential benefits of synthetic biology. Broadly speaking, two fields of application were mentioned by the participants. First, they believed that synthetic biology may contribute to greening the economy and combatting climate change. Second, their view was that synthetic biology may contribute to health and medicine, not least in developing countries. The strongest belief in the potential of synthetic biology was expressed by the Young Democrats, as illustrated by the following passage in their political pamphlet:

The world faces huge problems, and we need technology in our struggle against these problems. When one thinks of the world food deficiencies, our future energy supply and environmental pollution, synthetic biology will most likely play an important role in addressing these problems. An increasing population, climate change and a global biodiversity crisis means we cannot afford to lose any time, or exclude any innovation. – Young Democrats

The Young Democrats thus saw synthetic biology as an important instrument that can be used to address the grand challenges that the world is facing. The Young Socialists were also supportive of the developments and argued that synthetic biology should not be hindered by too many governmental constraints:

Because synthetic biology has such enormous potential it is important for the Netherlands to keep up or even take the lead in the field of research. The government should therefore not unnecessarily obstruct research with bureaucracy and let the decisions concerning synthetic biology be made by unbiased synthetic biology specialists. – Young Socialists

The Green PYO DWARS also recognized synthetic biology’s potential with regard to sustainability, which is that it could improve the production of biofuels and contribute to stopping climate change. Therefore, DWARS turned out to be fairly open to synthetic biology. This viewpoint is remarkable, because its mother party, GroenLinks, has a strong tradition of being against genetic modification (primarily in the food sector), deeming it a threat to biodiversity, ecological balances and the livelihoods of farmers in the global south [66]. In their ‘vision document’ [58], the extended Dutch version of the political pamphlet written for the MOYM, DWARS explicitly addresses this issue:

Within GroenLinks, genetic modification evokes the same sense of resistance as nuclear energy does. The commission however, does not intend to dismiss synthetic biology in advance. It proposes to explore where genetic modification fell short, and synthetic biology may contribute to the common good. Dismissing synthetic biology beforehand would constitute a missed opportunity and does not match the progressive nature of GroenLinks. Rather, a critical, resolute and sober approach is much more appropriate. In particular, because synthetic biology offers opportunities to save human lives. (author’s translation)^7^

This openness towards synthetic biology is in stark contrast with the Christian Democratic CDJA, which acknowledged the field’s potential regarding drug development and the production of renewable energy sources, but in the end remained highly critical. Correspondingly, the animal-welfare-inspired PYO PINK! doubted whether the field could solve the problems the world is facing and thought that it may even be a ‘cure’ that is worse than the disease:

Synthetic biology is a wonderful technology that may one day do wonderful things for us. But it’s just going too fast. Good technology plus bad policy equals bad outcomes. And we’re just not ready. We don’t even need this technology to solve many of the problems we currently face. Third world hunger is not a problem of food production, it is a problem of distribution. – PINK!

The Christian SGPJ and PerspectieF took a similar position, as can be illustrated by this quote from their joint political pamphlet:

Of course, technology can prove very helpful in tackling severe problems, but if we limit our worries exclusively to a technical solution, our lack of control about reality and the tendencies towards evil will pop up automatically. Technology may help to face challenges, but problems are not technological, but immaterial. – SGPJ/PerspectieF

Although the participants were often outspoken on what synthetic biology has to offer, they were also puzzled by some issues. The representative of the iGEM team of the University of Leuven, for instance, raised a tough issue by proposing the use of GMOs that stimulate the growth of new ice caps at the North Pole. Consideration of the appropriate intellectual property regime for synthetic biology also challenged the participants and the audience. The majority of the audience seemed to favour an open source regime, which became apparent after a quick poll by the moderator. This was not surprising since the audience consisted primarily of iGEM participants and the competition leans heavily on open source information. In response to this, the importance of patenting was underscored by one of the iGEM representatives:

But do you understand why there’s open source in the first place? Because in the case of IT, you need a couple of hundred dollars or a couple of thousand dollars to buy a computer and off you go. It’s totally different with synthetic biology. It’s all about multi-million dollar facilities. We’re talking about billions of research dollars being put at stake here. All people think of patents as a way of how companies can use it to leverage and profit from, but to be honest from a scientific point of view we need patents to protect our interests. – iGEM University College London

In their political pamphlet, the Young Socialists encourage open source initiatives but also uphold the possibility of patenting innovations:

We stimulate institutions to share their discoveries without charging the people who use them. This will speed up the research in synthetic biology which can lead to useful and lifesaving applications. On the other hand, people should have the right to patent their synthetic biology discoveries, because they have a right to own intellectual property. – Young Socialists

During the MOYM debate, the institute thus heard several viewpoints on the innovation potential of synthetic biology. Primarily, the Young Democrats and Young Socialists argued that synthetic biology could offer important benefits for society, and even the Green PYO DWARS had quite a liberal attitude towards the field. On the contrary, PINK! and SGPJ/PerspectieF did not believe that we need synthetic biology to address important challenges and, as will be discussed next, thought that it may even be dangerous for society. For in spite of the possible benefits, like any other technology, synthetic biology is not without risks.

### Risk Discourse

The potential risks were a major factor in earlier debates on biotechnology in Europe and the Netherlands. At the MOYM, the potential risks of synthetic biology also played a big part in the discussion. What type of risks were perceived and by whom? How are risks weighed against the potential benefits? In line with the rich body of ELSI research on synthetic biology, two types of risks can be distinguished in the debate: *biosafety* risks relating to the potential unintended consequences for humans and the environment, and second, *biosecurity*, that is, risks relating to the potential abuse of synthetic biology [33].

The CDJA, similar to SGPJ/PerspectieF, turned out to be very concerned about the potential ecological risks and found religious and scientific reasons to be cautious about synthetic biology:

From a Christian democratic perspective we see it [nature] as God’s creation which we as stewards have to take care of for the next generation. For this hall of scientists, I guess you prefer the Darwinist approach. Also in the Darwinist approach, you regard it as an equilibrium that has come into balance during billions of years. … We have seen with technological advances in the past that small pollutions may have great consequences. … We don’t always oversee what those consequences will be so if you start changing, altering the very fundamentals of species in the form of DNA, in the form of even introducing practically new species into the environment … this may influence those delicate balances in ways we can’t comprehend; we may not be able to oversee in the further future. – CDJA

As we saw in the previous section, DWARS was fairly liberal towards synthetic biology, which can also be illustrated by the following quote from their political pamphlet:

While synthetic biology offers possibilities for society, there are also risks involved. DWARS believes that the possible risks should not surpass the potential benefits. However, the risks should be taken seriously. – DWARS

During the discussion, however, it became clear that the deliberate release of modified organisms was considered too dangerous:

We are willing to discuss healthcare issues … but we’re extremely reluctant to let products out in nature, in the environment because it might damage ecosystems. It might do a lot of damage that we can’t predict. Then we’re extremely reluctant and we’ll have to look at that situation very specific. – DWARS

Interestingly, it is often not the technology as such that primarily worries critical PYOs but the fact that humans—with their limitations—are in control of the technology. PINK!, for instance, expressed such concerns:

As an evolutionist myself I know that nature has many imperfections. But I think it would be unwise to think that humanity can simply change that. It might even have catastrophic consequences. And I say this because one of the most striking natural flaws is the human mind itself. … We have a bounded rationality … we all have a little inner Homer Simpson. Whenever we have to deal with a new technology we must ask ourselves whether we are able to deal with it in a safe manner. – PINK!

The CDJA representative, who was involved in research as well, expressed the concern that some scientists may indeed take potentially harmful risks:

Sometimes you get carried away by the huge potential of the discovery you make … and you don’t oversee other risks or the bigger picture. – CDJA

In addition to concerns about potential unintended consequences of the developments, there were concerns relating to biosecurity, since—unfortunately—synthetic biology could also be intentionally used to cause harm. Such biosecurity concerns were notably expressed by DWARS and SGPJ/PerspectieF:

We know synthetic biology can potentially come up with frightening consequences. We can’t exclude false positive expectations and we are sure that even the best of the best synthetic biology application won’t abolish evil. – SGPJ/PerspectieF

Similarly, the spokesperson of DWARS wondered:

What if indeed some people have wrong intentions, have the knowledge and have the tools to say commit bio-terror and that would be disastrous. So at that point I actually got really scared of the possibilities of synthetic biology. – DWARS

Biosecurity and biosafety concerns thus played an important part in the debate, but according to some participants, there are also risks involved in *not* using synthetic biology’s potential. The representative of the iGEM team from Imperial College London—the team that eventually won the European Grand Prize [67]—argued as follows:

For our project we looked at desertification and it is a fact that every day an area 1.5 times the size of Amsterdam turns into desert every single day. These things just happen; they are a fact. And then we have to face how do we actually return this to what it should be like and how do we conserve ecosystems. …. We’re seeing countries trying to implement things to combat climate change and it’s just not really working, is it. And I think that synthetic biology is one of those great areas that might enable us to actually do something about it and yes, I completely agree that it should be completely safety tested … but do we really want to bypass this great opportunity of being able to actually undo the damage we’ve done? – iGEM Imperial College London

The representative of iGEM Imperial College London had strong support from the Young Democrats, who argued:

There’s all sorts of issues with nature, with environment. … Things are getting out of hand. …We don’t know what exactly will happen if we do not act, but that doesn’t mean that we should go … for the status quo by definition. We should compare those two options, the option with biotechnology and the associated risks and the option without improvement and those associated risks, because there’s plenty of risks with that option as well. – Young Democrats

During the debate, biosafety and biosecurity risks were raised as issues by several participants. These risks led PYOs such as the CDJA, SGPJ/PerspectieF and PINK! to take a very cautious stance towards synthetic biology. But, other voices stated that *not* using the potential of synthetic biology is also risky, given the grand challenges that societies are facing with regard to, for instance, the environment and climate change.

### Ethics Discourse

From the start, the (academic) debate about synthetic biology focused not only on risk–benefit aspects but also on issues relating to moral boundaries that perhaps should not be overstepped. Will synthetic biology lead to synthetic life one day? Will developments in the field lead to the ‘computerization’ of life and will this in turn diminish the definition of life [7, 8, 29]? Are synthetic biologists playing God [68]? Such ethical considerations also played a role in the MOYM debate.

In their joint political pamphlet, the Christian PYOs SGPJ and PerspectieF specifically address the notion of ‘humans as creators’:

Our ancestors believed they were part of creation; in our time we tend to think we are creators ourselves. The application of synthetic biology is a clear example where scientists see themselves as creators. As Christian politicians, we firstly want to express our recognition of God as Creator of all. This notion has a significant impact on our thinking about synthetic biology. As humans we have the Biblical mandate to ‘cultivate and preserve’ God’s creation. This means we have to benefit from opportunities and talents to do research and make new things. At the same time, preservation implicates reflection and long-term thinking. Such notions make synthetic biology an ethical issue. – SPGJ/PerspectieF

According to SGPJ/PerspectieF, synthetic biologists can thus be seen as ‘creators’, which therefore makes synthetic biology a theological matter. However, given the lack of thorough Christian reflection on synthetic biology, the two PYOs were hesitant to make a definitive judgement on how Christianity should deal with this issue and pleaded for more contemplation:

Until now there has not been a significant, broadly agreed Christian reflection on synthetic biology. Despite offering very valuable guidelines for scientists and politicians, the Bible doesn’t give a clear go or no-go for synthetic biology. In addition, there are several applications with different motives possible. … It’s very premature to connect Bible verses to BioBricks. – SPGJ/PerspectieF

As mentioned before, the CDJA acknowledged the opportunities synthetic biology may bring. According to their resolution [57] (which was based on their political pamphlet), the development of medical treatments is important and neglecting to carry out such development might be morally objectionable. On the other hand, so would tinkering with the building blocks of life. In this context, the CDJA believes that the development of synthetic biology may be at odds with the intrinsic value of nature and living beings:

Another important principle is the notion of transcendence. We can never rule out – neither scientifically nor philosophically – that nature is comprised of more dimensions and aspects than can be perceived by mankind. Therefore, it is unacceptable for Christian Democrats to consider and treat nature merely as a machine. Nature has intrinsic value. – CDJA (author’s translation)

The animal-welfare-inspired PYO PINK! added a similar sort of critique. Their representative denounced the hubristic aspirations of improving nature:

Do we really know how to perfect nature? In our arrogance in creating our own improved kind of nature we don’t seem to recognize how much conventional nature has to offer. Many of our medicines, for instance, are provided to us by nature. But we are destroying our natural capital at an immense rate. Before we start creating nature 2.0 it is high time we first truly value the original. – PINK!

Several iGEM teams responded to these remarks. First of all, they referred to synthetic biology’s potential to undo the damage that humankind has done to nature. Second, they denounced the alleged aspirations to perfect nature:

If you study nature you’re always in awe of what nature does …, so I personally would not assume that I can make nature perfect. I think what we are doing is quite ’crap’ compared to what nature invented in the last 3.5 billion years. It’s just a very tiny piece of what we can do. And so I personally feel humble to what nature does and what nature presents to me. – iGEM University of Freiburg

Coming back to the PYOs, the Young Democrats introduced a very different moral perspective on the developments, which became clear during the discussion on ownership:

Often life is defined by three basic properties: metabolism, growth and reproduction. And if you have these three basic properties then you can call something alive. … modern biology shows us that all these properties are defined by molecules, … so it is essentially a nanomachine. Essentially it is all a physical reaction. … Life in itself does not have a special moral status different from other machines. And that is what led us to our statement: a cell is a mere machine that we can create and modify. – Young Democrats

Much to the relief of PINK!, this did not imply that there can be other reasons why living beings are worthy of protection:

I don’t mean to say that all life is a mere machine. I’m just saying the phenomenon life doesn’t give something a special moral status. But for instance – and you [the representative of PINK!] shall be happy to hear – an animal, a sentient being or a human, they have certain protection. They are not being owned or are not being patented. But we do think that from this analysis logically it would follow that, if you have created life successfully in the lab, and we know that we are not near that yet, that you should be able to patent that as it is an invention. – Young Democrats

DWARS also considered the extent to which synthetic biologists are interfering with nature but eventually concluded that the subject matter should be approached in a more pragmatic way:

From a philosophical or religious perspective one could wonder if man should be allowed to alter life itself. From an ethical perspective one wonders how far these alterations should be allowed to go. Aside from one’s perspective, synthetic biology developments cannot be stopped. The question should not be if we want to use synthetic biology, but in what way. – DWARS

Lastly, the Young Socialists recognized that synthetic biology certainly raises ethical issues and advocated discussing them on a case-by-case basis, but they were less concerned about them than the other PYOs. Moreover, they believed that the public might be less worried about such ethical issues than expected by some:

80 years ago, abortion was something you couldn’t speak about, well only maybe in America still, but in Europe it’s weird to say I’m against abortion. I think that this will also be the case for synthetic biology. And so I think you really have to look at the public opinion of the time. – Young Socialists

According to the Young Socialists, techno-moral change [cf. 69] will therefore occur and will most likely be in favour of synthetic biology research. Despite their differing views, the participating iGEM teams did demonstrate great willingness to take ethical concerns seriously. According to the spokesperson of the iGEM team from Paris Descartes University, this willingness can be found throughout the entire community:

The Paris team has been looking at the human practices of the previous teams and we could see that ethics is a major concern in synthetic biology and most teams want to address these problems. – iGEM Paris Descartes University

In conclusion, during the MOYM debate, various moral issues were raised. Overall, it was vividly clear that for some groups in society, synthetic biology does indeed raise value-laden questions about how far humankind ought to go in redesigning ‘life’ and ‘nature’. The institute also learned that PYOs may have highly conflicting viewpoints on how to deal with these moral questions. Evidently, these viewpoints would translate into differing policy choices. So, how should synthetic biology be dealt with eventually? This will be discussed in the next and final section on *power and control*.

### Power and Control Discourse

Synthetic biology may have important potential for innovation, but at the same time, it is not without risks and also raises broader ethical issues. How do we balance these issues? Who will benefit from synthetic biology and who will carry the burden? What should the government’s role be in this regard, or the public’s? In sum, who will determine how synthetic biology should develop and what conditions should be taken into account along the way? These are all questions that make up a discourse on *power and control*.

In the preceding section, I noted that DWARS considered synthetic biology as an unstoppable development that should be guided, rather than trying to prevent it:

We should not be afraid of the things we do not know. We should look at these new technologies and we should see what they can mean for us in society and we should use them in a safe and responsible manner. – DWARS

This viewpoint of DWARS seems to resonate quite well with the idea of responsible research and innovation (RRI), which aims to conduct early assessment and contribute to shaping research and development (R&D) processes so that they are aligned with societal values and needs [70]. In this regard, DWARS, however, also saw an important role for politics, as outlined in their political pamphlet:

DWARS encourages regulation of synthetic biology through politics. Self-regulation by science could create conflict of interest and could hamper public acceptance of synthetic biology. – DWARS

SGPJ/PerspectieF also advocated that synthetic biology should be heavily subjected to political control, not least because synthetic biologists would be too biased:

You should not give a blank cheque to scientists. … Politicians should be eager to control synthetic biology. Synthetic biologists should not take the lead in that reflection. Scientists are intensively driven by their curiosity and by the opportunities and advantages of their discoveries. You could say that it is their job to be biased towards the positive side. – SGPJ/PerspectieF

The CDJA, which was also critical of synthetic biology throughout the entire discussion, clearly agreed that synthetic biologists should not be allowed complete freedom. In its resolution on synthetic biology, it introduced another reason for this view, namely that of intergenerational justice [cf. 6] and stewardship:

This [stewardship] implies that one generation should not be allowed to impose its preferences on future generations. This principle has broad implications. In this regard, it is hard to assess the consequences of emerging biotechnologies. … Therefore, precaution is warranted against applications that may have long-term consequences for man and the environment. – CDJA (author’s translation)

This perspective did indeed translate into a rather restrictive regulatory proposal put forward by the CDJA:

We feel this is not a desirable development and we feel that we should govern it by exception. So prohibit it all and then per development decide whether you want to make an exception for this specific development or not. And that would be our main line. We would be willing to make an exception, for example for drug development for very critical diseases. – CDJA

In addition, the CDJA brought attention to the issue of public funding. Synthetic biology research is often publicly funded and therefore calls for timely political perspectives:

If you look at where the research takes place, it’s mostly publicly funded institutions. So, as a government and as politics, we don’t merely need to accept what is going on, we are actually for a large part funding what is going on and as such, we also need to have an opinion and merely accepting that it happens and letting it happen for me, is a bit too passive. – CDJA

DWARS remained unconvinced of meaningful regulation via funding schemes, since this would significantly limit science and private entities would still conduct research on synthetic biology. The Young Democrats agreed that this would limit science too much. According to the spokesperson of the iGEM of the University of Freiburg, the current regulations are already limiting the development of synthetic biology:

I don’t know all the laws by heart but I feel they [regulations] are too strict and too paranoid in many points. You are asked to perform security requirements that are completely useless in the lab for working with microorganisms for example. They are exposing you to a lot of extra work that is unnecessary and also expensive. It’s blocking the science. – iGEM University of Freiburg

The spokesperson was strongly supported by the representative of the Young Democrats, who stood up for scientific freedom and explicitly expressed faith in scientists:

We ended up concluding that on the one hand, you cannot sort of pre-empt every new development, you can’t know in advance what’s going to happen, so laws and legislations are necessarily going to lag behind; but I don’t think you should want to do that either. Because what we considered is that ultimately if you look at evil intentions creating bio-weapons and things like that, the best defence against that is transparency; it’s openness. Knowing what is going on in the field, that allows scientists, and we have faith in scientists. – Young Democrats

The Young Socialists also pleaded for freedom in research in synthetic biology and in other potentially dangerous technologies, as noted in their pamphlet:

Although synthetic biology research has potential dangerous consequences we should continue with synthetic biology research as well as research in other potential harmful technologies. Firstly, if we would stop with every technology which is potentially dangerous, we can stop with almost every technology. Second, we cherish academic freedom as a right and we want to keep it that way (with exceptions for some excesses). Third, although it has potential dangerous consequences, synthetic biology also offers many benefits. – The Young Socialists

The apparent lack of trust of some PYOs did not pass unnoticed by the iGEM teams. Against this backdrop, the representative of the iGEM team of the University of Freiburg shared an idea that aimed to increase trust, namely an oath for synthetic biologists [71]:

There is so much mistrust in science and in the scientists; if you trust doctors with your life, why don’t you trust the scientists? That gave us the idea that we should do something about this and build up some trust. We thought how do the doctors get their trust and they have the Hippocratic oath and I think that’s what we need for scientists too. We need some statement, what we are standing for. – iGEM University of Freiburg

Interestingly, explicit calls for public engagement also stemmed from iGEM. Perhaps, this attitude towards society will also contribute to the building of societal trust:

I really think it’s important … to achieve transparency and in the UK we do it through public engagement. It’s a political tool used to spread awareness. … When they are empowered with the knowledge, they can then formulate their own independent opinions. That is unbiased, coming from their own. It’s not influenced by a mother who tells you this, the media who tells you this or politicians. – iGEM University College London

The representative of the iGEM team from the University of Leuven also expressed the need to reach out to the broader public:

We learned that we want to inform people but most of us are just scientists and we are not trained enough to inform other people but we are really willing to. We are not just monsters creating bigger monsters or something. We really want to inform the public. – iGEM University of Leuven

Turning back to the PYOs, the institute heard several—occasionally opposing—viewpoints on the amount and timing of governmental regulation of synthetic biology. However, there was also agreement on what elements could contribute to proper governance of synthetic biology. There were pleas for some form of ethical deliberation on synthetic biology by means of ethical (advisory) committees that could assess the developments on a case-by-case basis. The Young Socialists, for instance, noted that:

It is important to look at every case individually. So don’t say, well, this area of science is good so it’s allowed and this area of science is bad so it is not allowed. We should take every case as an individual case because the technology is new. They will explore new things, and every case is different. – Young Socialists

DWARS took a similar position but added that it should be a multidisciplinary committee that certainly does not consist of only scientists. The CDJA was of course much more critical but in terms of governance also pleaded for such a committee:

You can of course decide to make a committee for more cellular things in which permissions and exceptions are given more easily: when it comes to vertebrates where you take a stricter ethical committee and where it comes to humans, you basically say no unless the government and parliament approves. – CDJA

In conclusion, issues relating to power and control played a crucial role during the MOYM debate. The discussion was, however, primarily focused on (traditional) legislative and executive power. Broader issues such as consumers’ responsibilities were not discussed. Perhaps this is not surprising, because politically engaged young people are likely to frame issues in such political terms. Nevertheless, the discussion showed several dissenting viewpoints on how synthetic biology should be dealt with, which might cause tensions as synthetic biology matures.

### Comparing PYOs with Their Affiliated Parties

In the previous sections, it became clear that the PYOs that participated in the MOYM have divergent views on synthetic biology. In this section, I will cautiously reflect on how the positions taken by the PYOs relate to the views of their mother parties. Since there has been no real political debate on synthetic biology in the Netherlands to the date of the writing of this article, comparison is only possible by considering positions taken towards earlier biotechnologies. Another limiting factor is that for the last decade, the debate has been dominated by discussions on GMOs in agriculture and the position of large biotechnology companies [cf. 65]. Nevertheless, making a brief comparison may provide a useful picture of the views as they relate to those of the mother parties, especially because synthetic biology may raise questions that cannot self-evidently be answered from established political ideologies.

SGPJ/PerspectieF were critical of synthetic biology. They were concerned about the potential risks but—while finding it difficult to make a definitive judgement—also expressed reservations from a Christian point of view. This position correlates with earlier views expressed by the SGP and ChristenUnie. According to SGP, advances in nanotechnology and biotechnology give rise to fundamental questions, such as ‘What is life?’. And while modern humankind lacks perspective on this question, SGP considers its view as clear and valuable: God is the creator of all life and natural boundaries therefore exist for a reason [72]. Similarly, the ChristenUnie also objects to interfering with God’s creation [65, 73].

Furthermore, the Christian CDJA contested the developments in synthetic biology as well. It believed that synthetic biology should be banned in general but may be allowed in exceptional cases. This corresponds with the CDA’s view on animal biotechnology. On the other hand, the CDA has been a strong supporter of (agro-)biotechnology applications in the growing of crops [65, 74]. The critical stance of the CDJA towards synthetic biology is therefore not self-evident. The fact that synthetic biology allows deeper interventions in organisms compared to earlier biotechnology may be an important factor in this regard.

Just like the PYOs just mentioned, PINK!, related to the Dutch animal welfare party, the Partij voor de Dieren, was concerned about synthetic biology. The Partij voor de Dieren has been very critical of earlier biotechnologies, in relation to both animals and plants. According to the Partij voor de Dieren, genetic modification constitutes an unjustifiable violation of the genetic integrity of animals and plants and poses severe ecological risks [75, 76]. The views of PINK! therefore correspond to the views of the Partij voor de Dieren.

Turning to the PYOs that were more liberal towards synthetic biology, the Green-oriented DWARS turned out to be fairly open to synthetic biology. As I already outlined in the section on *innovation*, its mother party, GroenLinks, has a strong tradition of being against genetic modification [65, 66]. While noting that genetic modification was mostly met with resistance by its members, DWARS, however, proposed exploring where genetic modification fell short and believed that synthetic biology may contribute to the common good, such as improving health and combatting climate change. In comparing the PYOs and their mother parties, this is perhaps the most striking deviation.

The Young Socialists, related to the Dutch Labour party, the Partij voor de Arbeid (PvdA), seemed even more liberal towards synthetic biology than DWARS. According to their organization, the Netherlands should strive to reach a leading position in the field of research and the government should not unnecessarily obstruct research with bureaucracy. In comparison with the viewpoints of the PvdA on earlier biotechnologies, this seems more liberal. In the past decade, the PvdA has voted in favour of biotechnology developments on several occasions, but has always employed a case-by-case approach to biotechnology applications [65] and thus has objected to certain applications [cf. 77]. As discussed in the section on *power and control*, the Young Socialists also advocated a case-by-case approach, but expressed great enthusiasm towards synthetic biology.

The Young Democrats also welcomed the developments and repeatedly stressed synthetic biology’s potential to address urgent challenges. This position correlates with the supportive views of its mother party, D66. For instance, in its 2014 election programme for the European parliament elections, it notes that agro-biotechnology should be seen as an opportunity and restrictions should be lifted [78].

In conclusion, most of the PYOs’ positions seemed to be in harmony with viewpoints expressed earlier by their mother parties. However, the Young Socialists seemed to be more liberal than the PvdA. Furthermore, the CDJA was very critical of synthetic biology throughout the debate. This view compared to that of the CDA is interesting, because traditionally, the party has been very reluctant to accept animal biotechnology but has supported the use of biotechnology in crop agriculture The most remarkable deviation from party tradition was the openness of DWARS towards synthetic biology, since its mother party, GroenLinks, has objected to genetic modification fiercely in the past.

## Discussion

By organizing the MOYM debate, the Rathenau Instituut aimed to broaden the debate on synthetic biology early on in its development. As mentioned in the introduction, this activity corresponds with the shift in the focus of TA towards more early engagement in the last 10 years and the move upstream in the innovation process [11–13]. The idea of a MOYM was evidently sparked by the fact that the European–African Jamboree was held in the Netherlands. Nevertheless, this experimental event seemed to be a successful attempt to broaden the debate on synthetic biology and support mutual learning and understanding among the participants. First, the positive outcome was enabled by the open culture of iGEM towards society and societal issues, thus underscoring the on-going relevance of iGEM for RRI and TA. To quote one of the iGEM participants’ closing statement: ‘I think that this kind of debate is very useful in assessing where everyone in society is and synthetic biology should definitely be kept going in this kind of style’. Second, PYOs turned out to be valuable stakeholders to involve early on in a discussion about synthetic biology.

Given the positive outcome of the debate and the shift of TA towards new and emerging science and technology, collaboration with PYOs may be worth further consideration. In a broader perspective, collaboration with PYOs demonstrated that addressing political parties—rather than parliament itself—may be sensible for TA and RRI practitioners. In this regard, a political party should not be seen as a unity but as a multitude of organs and related bodies [79]. Several of these ‘political party-affiliated organizations’ could fulfil a valuable role in examining the potential significance of emerging technologies for the political party they are connected to. As well as applying to PYOs, this could apply to political think tanks (or scientific bureaus) and political working/advisory groups. Such organizations have not—to the best of my knowledge—been consulted (at least not prominently) in the practice of TA. In view of what was learned from the MOYM, I consider the potential value of engaging with political parties in TA and RRI activities a topic worthy of further research.

## Conclusion

For a couple of years now, the Rathenau Instituut has aimed to facilitate early political engagement with synthetic biology in the Netherlands. In this article, I discussed one of the activities that took place in this context: the MOYM. By fostering dialogue between future politicians, represented by Dutch PYOs, and future synthetic biologists, represented by iGEM participants, the institute aimed to broaden the debate on synthetic biology and support mutual learning and understanding among the participants. With regard to the latter, I would like to make the following remarks. On the one hand, the PYO representatives are not experts on synthetic biology, but they are used to debating in public. On the other hand, the iGEM representatives are experts on synthetic biology—in comparison—but they are not used to such public debates. So, both groups were both in and out of their respective comfort zone at the same time. Moreover, they treated each other with great respect. The spontaneous applause of the audience, consisting mostly of iGEM participants, after each comment—including the highly critical ones—is noteworthy.

In mapping the different viewpoints, several issues on which the PYOs disagreed with each other and the iGEM representatives could be found. In fact, several viewpoints seem irreconcilable. This is important, since the (pluralistic) composition of participants corresponds to (but does not speak on behalf of) different voices in society, such as the right wing, the left wing, Christians, Greens and those who are animal-welfare-oriented, etc. Having said that, the results of the debate are limited as well. The PYOs understood synthetic biology primarily from the perspective of (traditional political) legislative and executive power, thus leaving other societal perspectives (such as consumers’ responsibilities) unaddressed. Furthermore, the JOVD and ROOD did not participate, which is important because they are connected to big political parties. The JOVD’s mother party, VVD, even won the last two elections. In addition, the Party for Freedom is not affiliated with a PYO. Therefore, the perspectives of three important Dutch political parties were absent from the debate.

Nevertheless, given the absence of a comprehensive political debate on synthetic biology—and accordingly a lack of data on political viewpoints thereon—the discussion hopefully contributes to understanding where political challenges may arise. Accordingly, in conclusion, I will highlight four issues on which opposing viewpoints were taken and that therefore may be a potential source of political and societal tension:

- *The need for synthetic biology*
  During the MOYM, there were opposing viewpoints on how important synthetic biology can be for society. On the one hand, the Young Socialists, the Young Democrats and (to a lesser extent) DWARS advocated that society can benefit tremendously from the field. The Young Democrats—backed up by several iGEM representatives—even argued that there is risk in *not* using synthetic biology and that it can aid humankind in undoing damage it has done. On the other hand, PYOs such as SGPJ/PerspectieF and PINK! argued that we do not need synthetic biology to address the grand challenges that societies are facing. Focusing on the technology might only distract from real solutions and even pose unnecessary risks.
- *Concerns about deliberate release*
  Several envisaged applications of synthetic biology encompass the deliberate release of organisms into the environment. Consider, for instance, the use of GMOs to clean up plastic or oil spills in the ocean or to make desertified land fertile again [67]. During the MOYM, the majority of PYOs were very worried about such applications. PYOs such as PINK! and SGPJ/PerspectieF—that generally believe that synthetic biology is too complex to grasp and control—took this position. But, for a PYO such as DWARS—which was fairly liberal towards and optimistic about synthetic biology—deliberate release also seemed too dangerous a risk. On the basis of the MOYM, we can thus expect that these kinds of applications are likely to lead to intense debate as synthetic biology matures.
- *Moral boundaries*
  Whether it is the notion of humans as creators or the intrinsic value of life and nature that is important, the MOYM demonstrated that such notions play a real and important role for some PYOs and that synthetic biology is perceived to challenge these notions. Accordingly, they strongly influence how PYOs such as PINK!, the CDJA and SGPJ/PerspectieF understand synthetic biology and what policy choices they advocate. On the basis of the MOYM, it is not easy to predict when developments in synthetic biology cross the type of moral boundaries that should not be overstepped, but nevertheless, such concerns are likely to play an important role as the debate continues.
- *Political control*
  Lastly, the MOYM demonstrated contrasting viewpoints on the extent to which politics and government should aim to control the development of synthetic biology. The element of trust plays an important role in this regard. On the one hand, PYOs such as the Young Democrats and the Young Socialists expressed great trust in synthetic biologists and stated that governmental interference would only obstruct innovation. On the other hand, the concerned PYOs were convinced that synthetic biologists should not be allowed unlimited freedom of action and that the development of synthetic biology should thus be subjected to stringent political and governmental control from an early stage.
  How exactly the Dutch debate will evolve in the future remains to be seen. By organizing the MOYM, the Rathenau Instituut aimed to contribute to timely political opinion-making on synthetic biology. The results of the MOYM demonstrate that balancing and reconciling the different viewpoints is likely to prove a major challenge and will have an impact on the development of synthetic biology in the Netherlands. Hopefully, the discussion at the MOYM has contributed to understanding what kinds of issues are likely to play a role and to helping stakeholders to anticipate them.

## Footnotes

1 In Dutch, this is *Leven als Bouwpakket*. An English translation of the book was published as a special issue of *NanoEthics* [29].

2 It is worthwhile noting that PYOs are by no means a Dutch phenomenon but can be found worldwide. In addition, many PYOs collaborate in European or international umbrella organizations [see, for example, 44].

3 The techno-moral vignettes can be found on the Rathenau Instituut’s website: www.rathenau.nl/SynBio.

4 During the MOYM debate, PerspectieF and SGPJ joined together and put forward one spokesperson to represent both PYOs. PerspectieF and SGPJ are affiliated with relatively small Christian parties that often collaborate in this fashion, such as in the city council of The Hague.

5 It should be noted that the MOYM debate was also discussed in the 2013 Rathenau Instituut report *Politiek over Leven* [59], written in Dutch. The aforementioned political pamphlets were included as an annex in the report. An updated English version of the report was published in 2014 [39]. The pamphlets were not included though. The examination in these reports differs from that in this article, however. Earlier, the key narratives in public debates on science and technology as identified by Macnaghten et al. [60] were used, in combination with a typology of basic attitudes towards science and technology, as charted by the Rathenau Instituut [61]. Nevertheless, I consider this article to have added value since the MOYM is discussed in more detail. The political pamphlets were, for instance, annexed but not included in the examination in the aforementioned reports. Second, this article provides more detail on the preparation and organization of the event.

6 The framework originally distinguishes *reflective ethics* and *lay morality* in order to distinguish the professional ethics discourse (e.g. ethics committees, academic debate) and the public discourse respectively. Since this actor perspective does not apply to the MOYM debate, these clusters are replaced by *broader ethical issues*.

7 All of the translated excerpts were translated from Dutch.
